# Clinical significance of initial treatment for peritoneal lavage cytology-positive gastric cancer: outcomes according to treatment strategy

**DOI:** 10.1186/s12957-022-02512-6

**Published:** 2022-02-15

**Authors:** Akikazu Yago, Shusuke Haruta, Masaki Ueno, Yusuke Ogawa, Hayato Shimoyama, Yu Ohkura, Harushi Udagawa

**Affiliations:** Department of Gastroenterological Surgery, Toranomon Hospital, 2-2-2 Toranomon, Minato-ku, Tokyo, 105-8470 Japan

**Keywords:** Cytology-positive, Gastric cancer, Treatment strategy, Prognosis, Conversion

## Abstract

**Background:**

Although patients with positive lavage cytology (CY1) are classified as having stage IV disease, long-term survival without other unresectable factors (P0CY1) has been reported. Conversion gastrectomy in patients with a change in cytology status after induction chemotherapy might improve survival, but appropriate treatment remains controversial. Here, we reviewed our experience in treating CY1 gastric cancer to evaluate the best treatment strategy.

**Methods:**

Clinical and pathological findings of patients with a diagnosis of P0CY1 gastric cancer at Toranomon Hospital between February 2006 and April 2019 were retrospectively analyzed. Patients were classified into two groups according to initial treatment: a surgery-first group and a chemotherapy-first group. In addition, the patients were categorized into subgroups based on the subsequent treatment pattern. The surgery-first group was divided into two subgroups: adjuvant chemotherapy and palliative gastrectomy only. The chemotherapy-first group was divided into three subgroups with the subsequent treatment pattern depending on the response to chemotherapy: conversion gastrectomy, palliative gastrectomy after induction therapy, and palliative chemotherapy.

**Results:**

In total, 38 patients were eligible for inclusion in this study. After initial assessment of cytology status, 21 patients underwent gastrectomy as initial treatment (surgery first) and 17 received induction chemotherapy (chemotherapy first). Ten patients underwent surgery first with adjuvant chemotherapy, 11 underwent palliative gastrectomy alone, 5 underwent conversion surgery, 5 with CY1 disease after induction chemotherapy underwent palliative gastrectomy, and 7 received palliative chemotherapy only. The 3-year survival rate was 23.4% (median survival, 17.7 months) in the surgery-first group and 27.3% (median survival, 19.7 months) in the chemotherapy-first group. The 3-year survival rate was 75% for conversion gastrectomy, 16.7% for palliative chemotherapy, and 0% for palliative gastrectomy after induction chemotherapy.

**Conclusions:**

There was no significant difference in outcome according to whether surgery or chemotherapy was performed first. The prognosis of conversion surgery with curative resection was better than that of the other types of treatment. However, the outlook after induction chemotherapy was poor. Patients with advanced gastric cancer should be treated cautiously until more effective treatment options become available.

## Background

Patients with advanced gastric cancer usually undergo curative intent gastrectomy with adjuvant chemotherapy. Although survival in patients with metastatic gastric cancer has improved, it is still unsatisfactory [1, 2]. In the Japanese classification of gastric cancer, positive peritoneal lavage cytology (CY1) is defined as distant metastasis [1] and is the most significant predictor of poor survival after macroscopic curative resection [2]. However, there are several reports suggesting that survival is better in patients with positive cytology without other unresectable factors (P0CY1) than in those with macroscopic peritoneal dissemination [3, 4].

How best to treat patients with CY1 gastric cancer remains controversial. It has been reported that surgery combined with adjuvant chemotherapy may have a survival benefit in patients with CY1 gastric cancer, and S-1-based chemotherapy is usually administered following gastrectomy [5]. However, several recent reports show that a change from positive to negative cytology status following chemotherapy or being able to perform conversion surgery can improve the survival rate [6]. In our department too, we had performed not only gastrectomy with postoperative chemotherapy but also induction chemotherapy before surgery to aim for conversion gastrectomy.

In this study, we reviewed our clinical experience of the treatment outcomes and any subsequent problems encountered in patients with CY1 gastric cancer to evaluate an appropriate therapeutic strategy.

## Methods

### Study population

Clinical and pathological findings in patients who underwent gastrectomy and/or diagnostic laparoscopy in the Department of Gastroenterological Surgery, Toranomon Hospital, Tokyo, between February 2006 and April 2019 were reviewed through to March 2021. The study inclusion criterion was histologically proven gastric adenocarcinoma diagnosed as P0CY1 at the first assessment. Tumor stage and histopathological grade were categorized according to the 3rd edition of the Japanese Classification of Gastric Carcinoma [1]. Information on age, sex, surgical procedure, macroscopic appearance, histologic type, tumor size, depth of invasion, and lymph node metastasis were collected.

### Surgical procedure and cytology assessment

After confirming that there was no peritoneal dissemination by laparotomy or diagnostic laparoscopy, 100 ml of normal saline was injected into the pouch of Douglas and intraoperative peritoneal lavage cytology was performed. Gastrectomy with D2 lymph node dissection was usually performed for CY1 gastric cancer, sometimes with limited lymph node dissection. Achievement of macroscopic curative resection without residual tumor was confirmed in cases undergoing gastrectomy.

### Treatment plan

When the P0CY1 gastric cancer was diagnosed, an appropriate treatment plan was implemented at the discretion of the treating surgeons, with reference to the Japanese guidelines for the treatment of gastric cancer [7] and the latest developments in chemotherapy during the study period. Adjuvant and induction chemotherapy were performed depending on patient characteristics, such as general condition, tolerance of treatment, and adverse effects. In patients who underwent induction chemotherapy, cytology on diagnostic laparoscopy was reassessed after 2 or 3 courses of chemotherapy, except for cases with clear disease progression found on imaging. Subsequent treatment was also decided depending on patient characteristics.

### Treatment patterns

The patients were divided into a surgery-first group and a chemotherapy-first group according to the initial treatment provided after diagnosis of P0CY1 cancer. Patients in the surgery-first group were categorized further according to whether they received adjuvant chemotherapy or palliative gastrectomy alone. Patients were deemed not to have received maintenance chemotherapy postoperatively if there was no record of chemotherapy being introduced or if S-1, for example, could not be administered adequately for reasons other than disease progression. Patients in the chemotherapy-first group were further divided into a conversion surgery group that achieved negative cytology (CY0) and underwent curative resection, a palliative surgery group that underwent surgery but continued to be CY1, and a palliative chemotherapy group that continued on chemotherapy without a change to CY0.

### Chemotherapy

In accordance with the Japanese guidelines for the treatment of gastric cancer [7], patients received adjuvant chemotherapy consisting of S-1 administered orally at a dosage of 40 mg/m^2^ twice daily for 4 weeks followed by 2 weeks of rest or for 2 weeks followed by 1 week of rest as a single course. The chemotherapy regimens administered as induction therapy and/or palliative chemotherapy for progressive or unresectable gastric cancer were as follows: S-1 plus cisplatin (intravenous cisplatin 60 mg/m^2^ [day 8] followed by oral S-1 twice daily for 3 consecutive weeks followed by a 2-week rest), S-1 plus oxaliplatin (intravenous oxaliplatin 100 mg/m^2^ [day 1] followed by oral S-1 twice daily for 2 consecutive weeks followed by a 1-week rest), capecitabine plus oxaliplatin (intravenous oxaliplatin 130 mg/m^2^ [day 1] followed by oral capecitabine 1000 mg/m^2^ twice daily for 2 consecutive weeks followed by a 1-week rest), weekly paclitaxel (intravenous paclitaxel 80 mg/m^2^ weekly for 3 weeks followed by a 1-week rest), and other less frequently used regimens. All regimens followed the Japanese gastric cancer guidelines or regimens used in clinical trials in which patients were enrolled after informed consent was obtained. Computed tomography and upper gastroenterological endoscopy were performed after 2 or 3 cycles of chemotherapy to evaluate the clinical response of the target lesions.

### Statistical analysis

The primary endpoint of the study was overall survival, defined as the number of days patients survived after their initial surgery or induction of chemotherapy. Differences between the two groups were assessed using the chi-squared test or the unpaired Mann-Whitney *U* test. Predictors of overall survival and median survival time (MST) were examined by Kaplan-Meier survival curve analysis and compared using log-rank statistics. Marginally significant covariates were set to a *p* value of < 0.05. The *p* values for the primary analyses of overall survival and recurrence-free survival were one-sided while all other p-values were two-sided. All statistical analyses were performed using IBM SPSS Statistics version 23 (IBM Corp., Armonk, NY).

## Results

### Patient characteristics

**Fig. 1:**
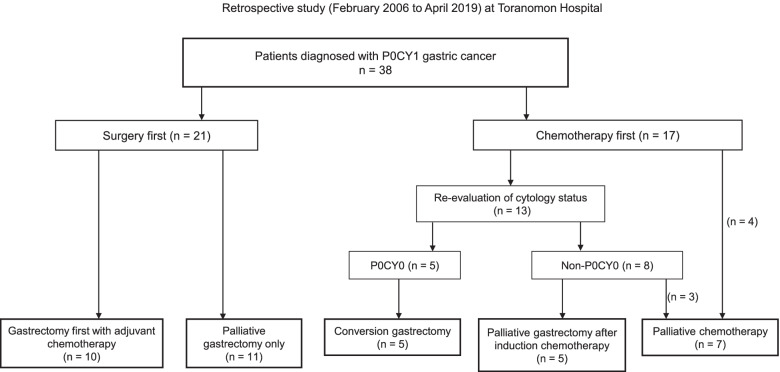
Flow diagram of patients in this study. Patients were diagnosed with P0CY1 gastric cancer at Toranomon Hospital between February 2006 and April 2019

Thirty-eight patients were diagnosed with CY1 gastric cancer without other unresectable features such as peritoneal dissemination at Toranomon Hospital between February 2006 and April 2019 and were eligible for inclusion in this study. Figure 1 shows the flow of patients through the study. After the initial assessment of cytology status, 21 patients underwent gastrectomy as their initial treatment (surgery first), and 17 received induction chemotherapy (chemotherapy first). Ten patients underwent surgery first with adjuvant chemotherapy, 11 underwent palliative gastrectomy alone, 5 underwent conversion surgery, 5 with CY1 disease after induction chemotherapy underwent palliative gastrectomy, and 7 received palliative chemotherapy only.

Table 1 shows the clinicopathological characteristics of the 38 patients according to whether surgery or chemotherapy was the initial treatment. No significant differences were observed between the two groups.

**Table 1:** Clinicopathological features of patients with advanced gastric cancer according to initial treatment

Variable		Surgery first *n* = 21	Chemotherapy first *n* = 17	*p* value
Age (years)		62 (32–92)	71.5 (41–80)	0.307
Sex	Male	14	11	0.899
	Female	7	6	
Main location	U	5	8	0.203
	M	12	5	
	L	4	4	
Macroscopic appearance	Type 2	4	1	0.483
	Type 3	9	8	
	Type 4	8	8	
Histologic type	Differentiated	3	5	0.255
	Undifferentiated	18	12	
c-Depth of invasion	SS	3	4	0.537
	SE	16	12	
	SI	2	1	
c-Lymph node metastasis	N0	7	3	0.616
	N1	7	6	
	N2	6	7	
	N3	1	1	

*L* lower, *M* middle, *SE* invasion of serosa, *SI* invasion of adjacent structures, *SS* invasion of subserosa, *U* upper

Table 2 shows the surgical and pathological characteristics of the 38 patients according to the treatment category. Total gastrectomy was performed in 23 patients (74.2%) and limited lymph node dissection was performed in 17 (54.8%). Table 2 also presents the pathological details in each category; 26 patients (83.9%) had pT4 disease and 20 (64.5%) had pN3 disease. Notably, 80% of patients who underwent conversion surgery had no detectable lymph node metastasis.

**Table 2:** Surgical and pathological characteristics according to treatment category

Variable		Gastrectomy with adjuvant chemotherapy *n* = 10	Palliative gastrectomy alone *n* = 11	Conversion gastrectomy *n* = 5	Palliative gastrectomy after induction chemotherapy *n* = 5
Surgical procedure	TG	5	9	5	4
	DG	5	2	0	1
Lymphadenectomy	D2	4	5	4	1
	Limited	6	6	1	4
Combined resection	Spleen Pancreas	2	3 2	1	-
Tumor diameter (mm)		77.5 (40–175)	122 (20–235)	133 (25–150)	97 (50–130)
Operating time (min)		318.5 (200–493)	402 (148–614)	338 (272–429)	346 (138–400)
Blood loss (ml)		356 (113–689)	494.5 (149–1897)	492 (269–770)	545 (215–820)
p-Depth of invasion	SS	0	0	3	2
	SE	9	9	2	3
	SI	1	2	0	0
p-Lymph node metastasis	N0	1	0	4	0
	N1	2	0	0	0
	N2	0	1	1	2
	N3	7	10	0	3

*DG* distal gastrectomy, *SE* invasion of serosa, *SI* invasion of adjacent structures, *SS* invasion of subserosa, *TG* total gastrectomy

The first-line chemotherapy regimens are shown in Table 3. The most commonly used regimen was based on S-1. In the group that underwent conversion surgery, 3 or 4 cycles of S-1 plus cisplatin or four or 6 cycles of S-1 plus oxaliplatin were administered before a re-evaluation of cytology status.

**Table 3:** First-line chemotherapy regimens for P0CY1 gastric cancer according to treatment category

	Gastrectomy with adjuvant chemotherapy *n* = 10	Conversion gastrectomy *n* = 5	Palliative gastrectomy after induction chemotherapy *n* = 5	Palliative chemotherapy *n* = 7
SP	5	3	3	3
SOX	2	2	1	1
S-1	2	0	0	1
XELOX	1	0	1	1
wPTX	0	0	0	1

*SOX* S-1 plus oxaliplatin, *SP* S-1 plus cisplatin, *wPTX* weekly paclitaxel, *XELOX* capecitabine plus oxaliplatin

### Patient survival

**Fig. 2:**
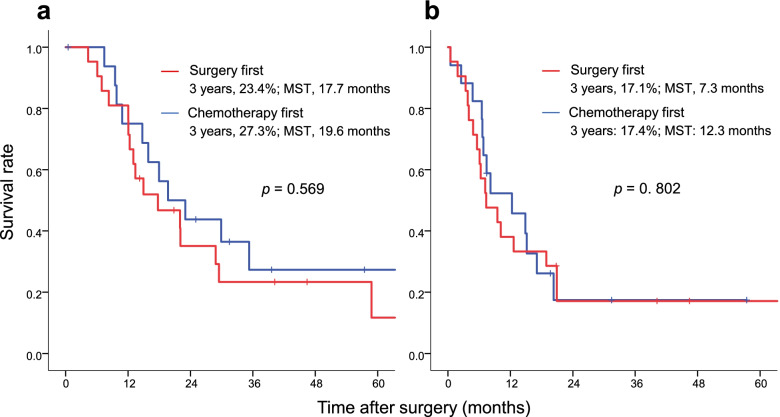
Kaplan-Meier curves for the surgery-first group and the chemotherapy first group. **a** Overall survival. **b** Recurrence-free survival. MST, median survival time

**Fig. 3:**
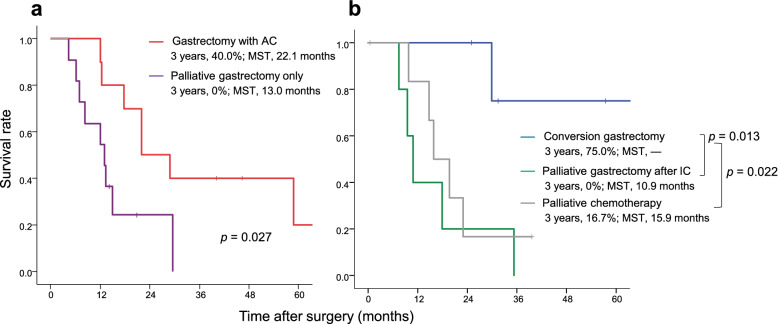
Comparison of the Kaplan-Meier curves for overall survival according to initial treatment. **a** Surgery first. **b** Chemotherapy first. AC, adjuvant chemotherapy; IC, induction chemotherapy; MST, median survival time

The overall survival curves according to initial treatment are shown in Fig. 2a. The 3-year survival rate was 23.4% (MST, 17.7 months) in the surgery-first group and 27.3% (MST, 19.7 months) in the chemotherapy-first group. The survival rate and MST in each treatment group are shown in Fig. 3. The 3-year survival rate was 75% in patients who underwent conversion gastrectomy, 16.7% in those who received palliative chemotherapy, and 0% in those who underwent palliative gastrectomy after induction chemotherapy.

### Patterns of recurrence

Recurrence-free survival according to initial treatment is shown in Fig. 2b. The 3-year recurrence-free survival rate was 17.1% (MST, 7.3 months) in the surgery-first group and 17.4% (MST, 12.3 months) in the chemotherapy-first group, respectively. Table 4 shows the recurrence pattern according to treatment category. There were 32 cases of recurrence; the most common of which was peritoneal dissemination (78.1%, *n* = 25), regardless of treatment category.

**Table 4:** Disease recurrence patterns according to treatment category

	Gastrectomy with adjuvant chemotherapy *n* = 10	Palliative gastrectomy alone *n* = 11	Conversion gastrectomy *n* = 5	Palliative gastrectomy after induction chemotherapy *n* = 5	Palliative chemotherapy *n* = 7
**Recurrence**	**7**	**10**	**3**	**5**	**7**
Peritoneal dissemination	4	10	3	4	4
Lymph node	2	0	0	1	2
Lung	1	0	0	0	0
Local recurrence	0	0	0	0	1

### Long-term survivors

The pathological characteristic of long-term survivors is shown in Table 5. Seven patients had over 3-year survival. Among them, gastrectomy with adjuvant chemotherapy had been performed in four patients, conversion gastrectomy in two patients, and palliative chemotherapy in one patient. Although there were no obvious differences in pathological features compared to non-long-term survivors, the tumor size tended to be smaller in long-term survivors.

**Table 5:** Clinicopathological features of 3-year survivors

Case	Category	Age	Sex	p-Depth of invasion	p-lymph node metastasis	Histology	Tumor diameter (mm)	Regimens of chemotherapy	Survival time (months)	Status
1	Gastrectomy with adjuvant chemotherapy	56	M	SE	N1	tub2	45	S-1, SP	81.2	Alive
2	Gastrectomy with adjuvant chemotherapy	32	M	SE	N3	sig	80	S-1	58.8	Dead
3	Gastrectomy with adjuvant chemotherapy	61	M	SE	N1	por	40	SOX, S-1	46.4	Alive
4	Gastrectomy with adjuvant chemotherapy	61	M	SE	N2	por	50	S-1	40.2	Alive
5	Conversion gastrectomy	57	F	SS	N0	por	133	SP	68.9	Dead
6	Conversion gastrectomy	74	M	SS	N0	tub1	25	SP	57.4	Alive
7	Palliative chemotherapy	67	F	cSE	NX	por	80	XELOX	39.6	Alive

*tub1* well-differentiated tubular adenocarcinoma, *tub2* moderately differentiated tubular adenocarcinoma, *por* poorly differentiated adenocarcinoma, *sig* signet ring cell carcinoma, *SOX* S-1 plus oxaliplatin, *SP* S-1 plus cisplatin, *XELOX* capecitabine plus oxaliplatin

## Discussion

This study investigated the effect of the initial treatment strategy selected on prognosis in patients with CY1 gastric cancer. We found no significant difference in the survival outcome according to whether the initial treatment was surgery or chemotherapy. However, subgroup analysis suggested that patients who underwent successful conversion surgery (curative resection after a change to CY0) tended to have a better prognosis than those who received alternative treatment. Patients who underwent surgery first followed by S-1-based maintenance chemotherapy postoperatively had the next best prognosis.

The administration of perioperative treatment is essential for advanced gastric cancer receiving surgery. Without proper perioperative chemotherapy, patients can be associated with a high rate of recurrence and metastases [8]. Chemotherapy for CY1 gastric cancer has been traditionally administered after gastrectomy. The prognosis in patients treated using this strategy is better than that in patients with other types of stage IV gastric cancer who receive adequate adjuvant chemotherapy and seems to be comparable with that in patients with stage III type 4 disease. Kodera et al. [3] reported that patients with CY1 gastric cancer had a 5-year survival rate of 26% and a recurrence-free survival rate of 21%, while Kano et al. [9] reported an MST of 15.9 months and a 5-year overall survival rate of 9.8%. Our 5-year survival rate in patients with CY1 gastric cancer was 11.7%, which is in line with these previous reports.

Poor prognostic factors should also be considered in patients with CY1 gastric cancer. It has been reported that patients with a high rate of lymph node metastasis or type 4 CY1 gastric cancer have a poor outlook [9–11]. It may be difficult to devise an appropriate treatment strategy for these patients. In such cases, we might have to balance some different points of view.

Several recent reports have shown that a change in cytology status from positive to negative following chemotherapy improves the survival rate [12, 13] and that conversion therapy may be a new and promising treatment in gastric cancer patients with CY1 or with peritoneal metastasis [14, 15]. However, clinicians are still faced with the problem of not being able to predict the response to chemotherapy or the pathological diagnosis before the initial treatment. Although conversion surgery with macroscopic curative resection would be expected to have a good prognosis, it was successful in only 5 of 17 patients in our study; this conversion rate of 29.4% cannot be considered successful, even in a relatively small number of cases. Moreover, the prognosis was dismal in patients with ongoing CY1 status and those with progressive (including unresectable) disease. In our study, the initial treatment choice was based on “when the response to chemotherapy is unknown.” Surgery first might be better for the patient if cytology does not turn negative. The strategy should be chosen keeping in mind that surgery may not be suitable for high-risk patients in whom the likelihood of efficacy is low.

It is not known whether our induction therapy strategy was appropriate. More intensive and/or persistent chemotherapy, for example, continuing until a change in cytology status to negative, may have improved the rate of conversion therapy and the prognosis. Various strategies are presently being used in an effort to improve the prognosis in these patients. If advances in induction therapy, including immuno-oncologic therapy, can increase the response rate, the outcome of a chemotherapy-first strategy might improve further. There have been reports on the usefulness of neoadjuvant or intraperitoneal chemotherapy followed by surgical resection [6, 11], and it was also reported that patients with limited peritoneal involvement might have a survival benefit from surgery with extended lymphadenectomy and R0 resection [16]. If these methods become useful as induction therapy for CY1 gastric cancer, there might be an improvement in the conversion rate and survival rate. Furthermore, it would be helpful to identify biomarkers that can predict recurrence. Tokuhisa et al. [17] identified several exosomal miRNA profiles in peritoneal fluid that might be useful as biomarkers of peritoneal recurrence after curative surgery in patients with gastric cancer. In the future, these biomarkers could be useful and help to determine the treatment strategy for CY1 gastric cancer.

This study had several limitations. First, it was based on limited retrospective analysis and contains a degree of selection bias in that there may have been some important reasons for performing surgery first, such as bleeding, stenosis, age, and patient wishes. These factors might have influenced the prognosis. Second, we did not perform diagnostic laparoscopy in all patients with gastric cancer; for example, patients who received neoadjuvant chemotherapy did not always undergo this procedure. CY1 cases might have been overlooked before starting neoadjuvant chemotherapy. Also, even though the usefulness of diagnostic laparoscopy for early-stage gastric adenocarcinoma has been reported [18], it was not included in this study. Third, the possibility of peritoneal dissemination in some cases cannot be excluded due to insufficiently detailed observation when diagnostic laparoscopy was performed. It is unlikely that multiple peritoneal dissemination would have been overlooked but it is possible that small nodules were missed. Although various issues remain to be resolved, we hope that appropriate assessment and decision-making before initial treatment will improve the prognosis in patients with CY1 gastric cancer.

## Conclusions

We found no significant difference in the prognosis of CY1 gastric cancer according to whether the initial treatment was surgery or chemotherapy. Patients who underwent surgery first followed by maintenance chemotherapy had a better prognosis and those who underwent conversion surgery with curative resection had an even better prognosis than those who received other types of treatment. However, the prognosis of CY1 that did not change with induction therapy was dismal. These findings may help clinicians with treatment selection in patients with cytology-positive advanced gastric cancer while we await development of more effective therapies.

## Data Availability

The datasets used and/or analyzed during the current study are available from the corresponding author on reasonable request.

## Abbreviations

MST: Median survival time
